# Draft Genome Sequence of the Halophilic Strain Citrobacter braakii AN-PRR1, Isolated from Rhizospheric Soil of Rice (Oryza sativa L.) from Pakistan

**DOI:** 10.1128/MRA.00787-21

**Published:** 2021-09-23

**Authors:** Aniqa Nawaz, Fathia Mubeen, Zia Ul Qamar, Muhammad Usama Marghoob, Saefuddin Aziz, Harald Gross

**Affiliations:** a Department of Pharmaceutical Biology, Pharmaceutical Institute, University of Tübingen, Tübingen, Germany; b Microbial Physiology Lab, Soil and Environmental Biotechnology Division, National Institute for Biotechnology and Genetic Engineering, Constituent College of Pakistan Institute of Engineering and Applied Sciences, Islamabad, Pakistan; c Department of Nuclear Institute of Agriculture and Biology, Constituent College of Pakistan Institute of Engineering and Applied Sciences, Islamabad, Pakistan; d Microbiology Department, Biology Faculty, Jenderal Soedirman University, Purwokerto, Indonesia; SIPBS, University of Strathclyde

## Abstract

Citrobacter braakii AN-PRR1 is a potential salt-tolerant, plant growth-promoting rice rhizobacterium isolated from Pakistani soil. The 4.9-Mb draft genome sequence contributes to its taxonomic classification and will reveal the genes putatively responsible for its osmoprotectant and plant growth-promoting activity.

## ANNOUNCEMENT

Rice, an important cereal worldwide, is under major threat from growing issues like salinity and drought due to the ongoing climate change (1–4). To combat the adverse effects of salinity on the production of rice and other crops, the use of salt-tolerant plant growth-promoting rhizobacteria (PGPR) is a promising approach (4–7). Therefore, we isolated and characterized salt-tolerant PGPR strains from rhizospheric soil of rice plants grown in salt-affected areas of Pindi Bhattiyan (31°53′52.12″N, 73°16′22.87″E), Hafizabad District, Punjab, Pakistan. Based on the screening process, isolate AN-PRR1 was prioritized, and its sequencing was initiated to clarify its taxonomic position, to reveal the genetic background of its characteristics, and finally to shed more light on the complete biosynthetic capacity for secondary metabolism.

Genomic DNA (gDNA) of AN-PRR1 was harvested from an overnight culture grown at 37°C in lysogeny broth (8) on a rotary shaker (220 rpm) and isolated as previously described (9). The gDNA (20 μg) was sheared using a Covaris g-TUBE device; libraries were constructed using the SMRTbell template preparation v1.0, Sequel binding v2.0, and MagBead v2 kits, followed by size selection using the BluePippin size selection system (Sage Science, Inc.). The 6-kb multiplex library was sequenced on a PacBio Sequel instrument and one SMRT cell. No quality filtering was conducted; however, subreads shorter than 50 bp were discarded. The remaining PacBio long reads were assembled using SMRT Link v7.0.1 and HGAP4 (10, 11). Default settings were used for all software, except for the HGAP4 genome size estimate parameter, which was set to 5 Mbp. Overall, the reads were assembled into one contig to construct a draft genome sequence. Since this genome resulted in a high number of pseudogenes (1,850 out of 4,592 coding DNA sequences [CDS]), mainly due to frame shifts and short indels, the gDNA extraction procedure was repeated, and upon library preparation (10 μg gDNA; Nextera XT paired-end library), the genome was resequenced using the Illumina NovaSeq 6000 platform. The initial quality assessment was based on data passing the Illumina Chastity filter. Subsequently, reads containing a PhiX control signal were removed. In addition, reads containing adapters were clipped. The second quality assessment was based on the remaining reads using the FASTQC tool v0.11.8 (12). Upon filtering, high-quality Illumina reads were assembled into contigs using ABySS v2.0.2 (13). The results of both sequencing platforms were subsequently used to perform a *de novo* hybrid assembly. The contigs were linked based on the alignment of the PacBio long reads. Alignment was performed using BLASR v1.3.1 (14). From the alignment, the orientation, order, and distance were determined using SSPACE-LongRead v1.0 (15). Using the Illumina reads, gapped regions within the contigs were closed using GapFiller v1.10 (16). Finally, assembly errors and the nucleotide disagreements between the Illumina reads and the PacBio-based sequences were corrected using Pilon v1.21 (17). The final genome sequence consists of two contigs and comprises 4.9 Mb. Genome annotation was conducted using the Prokaryotic Genome Annotation Pipeline (PGAP) v5.1 (18, 19). The genome features are summarized in Table 1.

**TABLE 1:** Sequencing metrics for C. braakii AN-PRR1

Statistic or characteristic	Data for *C. braakii* AN-PRR1
PacBio sequencing	
No. of reads	422,209
Mean read length (bp)	4,428
No. of mapped reads	417,958
Avg coverage (×)	348.95
No. of contigs	1
*N*_50_ (bp)	4,936,749
No. of gaps	0
Illumina sequencing	
Illumina read length (nt)	2 × 150
No. of read pairs	28,344,083
Yield (Mbp)	8,149
Avg quality	35.47
Avg coverage (×)	1,638.90
Insert size median (bp)	387.00
*De novo* hybrid assembly	
Genome size (bp)	4,929,517
GC content (%)	52.1
No. of contigs	2
*N*_50_ (bp)	4,102,519
No. of genes (total)	4,688
No. of genes (coding)	4,531

A genome-based taxonomic analysis of strain AN-PRR1, employing the Type Strain Genome Server (TYGS) (20), revealed that Citrobacter braakii ATCC 51113 represents the closest related type strain. In pairwise comparisons, independent of the applied Genome BLAST Distance Phylogeny formula, the digital DNA-DNA hybridization (dDDH) values *d*_0_, *d*_4_, and *d*_6_ ranged from 74.5 to 91.3% and were therefore within the species threshold of 70%. Thus, AN-PRR1 represents a Citrobacter braakii strain. Automated secondary metabolism analysis using antiSMASH v6.0.0 (21) predicted three biosynthetic gene clusters encoding a thiopeptide, a turnerbactin-like siderophore (22), and an arylpolyene (23).

### Data availability.

This whole-genome sequencing (WGS) project has been deposited at DDBJ/ENA/GenBank under the accession number JAGMWL000000000. The corresponding raw sequencing data set has been registered in the NCBI SRA database under the accession numbers SRX10639629 (PacBio) and SRX10639630 (Illumina).
